# Diabetes Management by Ayurvedic Practitioners Using a Clinical Guideline Versus Usual Practice: A Feasibility Cluster Randomized Trial in Nepal

**DOI:** 10.1155/jdr/2602864

**Published:** 2026-04-29

**Authors:** Kaushik Chattopadhyay, Shristi Karki, Haiquan Wang, Prerok Regmi, Pradip Gyanwali, Vasudev Upadhyay, Michael Heinrich, Bihungum Bista, Nikhil Tandon, Sanjay Kinra, Sheila Margaret Greenfield, Tuhin Kanti Biswas, Panniyammakal Jeemon, Jo Leonardi-Bee, Sarah Anne Lewis, Meghnath Dhimal

**Affiliations:** ^1^ Lifespan and Population Health, School of Medicine, University of Nottingham, Nottingham, UK, nottingham.ac.uk; ^2^ The Nottingham Centre for Evidence-Based Healthcare: A JBI Centre of Excellence, Nottingham, UK; ^3^ Nepal Health Research Council, Kathmandu, Nepal, nhrc.org.np; ^4^ School of Exercise and Health, Shanghai University of Sport, Shanghai, China, sus.edu.cn; ^5^ Department of Ayurveda and Alternative Medicine, Ministry of Health and Population, Kathmandu, Nepal, mohp.gov.np; ^6^ Centre for Pharmacognosy and Phytotherapy, School of Pharmacy, University College London, London, UK, ucl.ac.uk; ^7^ Chinese Medicine Research Center / Department of Chinese Pharmaceutical Sciences and Chinese Medicine Resources, College of Chinese Medicine, China Medical University, Taichung, Taiwan, cmu.edu.tw; ^8^ Department of Endocrinology, Metabolism and Diabetes, All India Institute of Medical Sciences, New Delhi, India, aiims.edu; ^9^ Department of Non-Communicable Disease Epidemiology, London School of Hygiene and Tropical Medicine, London, UK, lshtm.ac.uk; ^10^ Institute of Applied Health Research, University of Birmingham, Birmingham, UK, birmingham.ac.uk; ^11^ Department of Kayachikitsa, J.B. Roy State Ayurvedic Medical College and Hospital, Kolkata, India; ^12^ Sree Chitra Tirunal Institute for Medical Sciences and Technology, Trivandrum, India, sctimst.ac.in

**Keywords:** Ayurveda, clinical practice guideline, evidence-based, feasibility trial, management, Nepal, type 2 diabetes mellitus

## Abstract

**Background:**

Type 2 diabetes mellitus (T2DM) is prevalent in Nepal, with many seeking primary care through Ayurveda, a widely practiced traditional system. However, concerns exist about suboptimal care and variability in clinical practice among Ayurvedic practitioners. No evidence‐based clinical practice guideline (EB‐CPG) is available for managing T2DM. Therefore, an EB‐CPG was developed, and a feasibility study was conducted to inform a future cluster randomized controlled trial (RCT) assessing whether EB‐CPG improves T2DM management compared with usual practice.

**Methods:**

A two‐arm feasibility cluster RCT was conducted in Ayurveda centers. Centers were randomized (1:1) by an independent statistician. Adults with newly diagnosed, treatment‐naïve T2DM and glycated hemoglobin (HbA1c) of 6.5%–< 9% were recruited. Data collectors and the analyst were blinded to group allocation.

**Results:**

Fourteen Ayurveda centers were approached, all recruited (seven/group) and completed the study. One center withdrew and did not enroll participants. Of 151 potential participants, 121 (80%) were recruited (60 in intervention and 61 in control). Of those, 84% were followed up to 6 months (51/group). The median adherence score to EB‐CPG among practitioners was 1–2 (partial to adequate adherence). The median (interquartile range) number of EB‐CPG–recommended medicines not consumed by participants and days without consumption was 0 (0–10) and 0 (0–2.5), respectively. No serious adverse events occurred. Preliminary estimates suggest EB‐CPG′s beneficial effects on HbA1c, fasting plasma glucose, and health‐related quality‐of‐life, though not statistically significant.

**Conclusion:**

This feasibility trial demonstrated successful recruitment, follow‐up, and intervention adherence. A definitive trial is feasible to evaluate the intervention′s effectiveness in T2DM management.

**Trial Registration Number:**

ClinicalTrials.gov Identifier: NCT05259735; first posted on March 02, 2022.

## 1. Introduction

Type 2 diabetes mellitus (T2DM) is a widespread chronic condition that imposes a significant health and socioeconomic burden globally [1, 2]. Persistent hyperglycemia in this complex metabolic disorder is associated with a variety of complications, including macrovascular and microvascular issues, and even early mortality [1, 2]. In Nepal, the prevalence of T2DM increased from around 8% between 2010 and 2015 to 11% between 2015 and 2020 [3]. This may be an underestimation, as many people remain undiagnosed due to the often asymptomatic nature of T2DM in its early stages [4].

Ayurveda, the predominant traditional medical system in Nepal, has been practiced for thousands of years to address people′s primary healthcare needs [5]. Today, trained and registered Ayurvedic practitioners play a crucial role in public and private healthcare, often being the primary clinical care providers in Ayurveda centers [5, 6]. The fundamental approach to T2DM treatment is similar in both Western and Ayurvedic medical systems, emphasizing a combination of a healthy lifestyle and medicinal products [7]. T2DM is among the most common conditions for which people seek Ayurvedic care, with many individuals consistently using Ayurvedic medicines—composed of plant, animal, or mineral ingredients, either singly or in combination—from the time of diagnosis [6, 8]. This preference is largely shaped by cultural health beliefs and a desire to avoid the side effects, costs, and administration methods of Western medicines (e.g., insulin injections), particularly among rural, poor, older, and tribal communities, and the limited access to Western medicine physicians in remote areas [8–13].

Although systematic reviews of randomized controlled trials (RCTs) support the effectiveness and safety of Ayurvedic medicines in improving glycemic control and other vital outcomes in T2DM [14, 15], concerns persist about the suboptimal clinical care provided by Ayurvedic practitioners in real‐world settings [11, 16–21]. These concerns arise from the use of untested, ineffective, potentially unsafe, nonstandardized, or low‐quality Ayurvedic formulations, which may lead to serious adverse effects, including heavy metal toxicity [17, 21–25]. Many practitioners also depend on unverified claims or use a trial‐and‐error approach to treatment [20, 26]. Contradictory lifestyle advice is sometimes given, such as recommending ghee consumption (clarified butter made from saturated fat) [27]. Key steps in the care pathway—like detecting and managing poorly controlled T2DM and its complications, including specialist referrals—are often left to individual discretion, resulting in unacceptable variations in clinical practice [11, 13, 16, 17, 19–21].

Evidence‐based clinical practice guidelines (EB‐CPGs) contain recommendations grounded in the best available scientific evidence, designed to assist clinicians in decision‐making and ensure the delivery of quality care [28, 29]. Essentially, EB‐CPGs are instruments to bridge the gap between scientific research and clinical practice [28, 29]. Although CPGs in Western medicine have demonstrated improvements in T2DM management [30, 31], no such CPGs are available for Ayurvedic practitioners in Nepal. An EB‐CPG could potentially address the existing challenges, as several stakeholders, including these practitioners, have highlighted its absence as a major barrier [11, 20, 32–34]. To address this gap, we developed an EB‐CPG and conducted a feasibility study with the aim of informing a future definitive cluster RCT to assess whether the use of the EB‐CPG leads to better management of T2DM compared with the usual clinical practice (i.e., without a CPG) [35, 36]. Examining the feasibility of key elements before initiating a costly definitive trial improves its chances of successful completion and helps estimate essential parameters for trial design [37].

## 2. Methods

The study protocol, including all relevant details, is published elsewhere [36]. The study is reported adhering to the relevant Consolidated Standards of Reporting Trials (CONSORT) guidelines [38, 39].

### 2.1. Design

The study was a two‐arm feasibility cluster RCT.

### 2.2. Setting

The study was conducted in public and private Ayurveda centers (clusters), within and outside the Kathmandu Valley in Nepal, locally coordinated by the Kathmandu‐based Nepal Health Research Council (NHRC). Each center had at least one Ayurvedic practitioner, with at least a 5½‐year undergraduate medical degree in Ayurveda and registered with the Nepal Ayurveda Medical Council.

### 2.3. Randomization and Blinding

Ayurveda centers willing to participate in the feasibility trial were randomized (1:1) to either intervention or control group, by an independent statistician based at NHRC using a computer‐generated randomization schedule. Although Ayurvedic practitioners and participants (patients) could not be blinded to group allocation, data collectors (outcome assessors) and the data analyst (statistician) were blinded. To avoid contamination between treatment groups, randomization was at the center level, and additionally, intervention group practitioners signed an intervention–nondisclosure agreement.

### 2.4. Intervention

Ayurvedic practitioners utilized the EB‐CPG developed by our team to manage T2DM, available in both hard and soft copies [35]. In brief, we developed a high‐quality, comprehensive EB‐CPG for T2DM management using a robust methodology that incorporated the best available scientific evidence and engaged key stakeholders, including practitioners. The EB‐CPG addresses topics such as T2DM diagnosis and treatment targets, lifestyle guidance, Ayurvedic antidiabetic medicines, screening and management of complications, and referrals to specialists for poorly controlled T2DM and its complications. To facilitate its adoption and adherence, practitioners participated in regular in‐person and online group training on using the EB‐CPG, including individual role‐playing and feedback and peer‐led sessions to consolidate learning. They utilized an EB‐CPG–based structured booklet for recording case notes. Furthermore, a standardized, quality‐controlled, plant‐based Ayurvedic antidiabetic capsule—recommended by the EB‐CPG—was made available at the Ayurveda centers and provided free of charge to participants throughout the study.

### 2.5. Control

The usual clinical practice continued, where Ayurvedic practitioners managed T2DM without using a CPG.

### 2.6. Participant Eligibility, Recruitment, and Follow‐Up

Individuals from diverse socioeconomic backgrounds visit the Ayurveda centers, primarily for symptom‐related concerns, with T2DM often identified incidentally. When participating Ayurvedic practitioners diagnosed new, treatment‐naïve T2DM cases, these individuals were approached for trial participation.

The participant information sheet (available in Nepali and English) was provided to potential participants. A verbal study description was also given, and any questions were answered. Those providing written informed consent (available in Nepali and English) were assessed against the study′s eligibility criteria. We included adults (≥ 18 years) with glycated hemoglobin (HbA1c) ranging from 6.5% [40] to < 9% (for safety reasons) and excluded people who were pregnant, had any serious medical condition or were terminally ill (e.g., cancer), or were receiving any related nonpharmaceutical or pharmaceutical interventions. All eligible people were recruited over the recruitment period (from July 17, 2022 to July 4, 2023) and followed up for 6 months.

### 2.7. Study Parameters and Data Collection

The main feasibility outcomes were trial recruitment, trial follow‐up, and intervention adherence. More specifically, these included the recruitment of Ayurveda centers and participants, follow‐up of participants, adherence to the EB‐CPG among intervention group Ayurvedic practitioners (through written case notes review of all the intervention participants at the end of their trial participation), and compliance with the EB‐CPG–recommended Ayurvedic antidiabetic medicine among intervention group participants (through participant self‐reported diaries and capsule counting at centers). Additionally, we also explored the following: Ayurvedic medicines prescribed by practitioners in the control group (through written case notes review of all the control participants at the end of their trial participation) and participants′ characteristics, effectiveness outcomes (including key effectiveness outcomes such as HbA1c, fasting plasma glucose, and health‐related quality‐of‐life [HRQoL]), and safety (including adverse events [AEs] and serious adverse events [SAEs]). Data collection, including blood and urine samples, followed standardized operating procedures, with samples analyzed at local laboratories accredited by Nepal′s National Public Health Laboratory.

### 2.8. Data Analyses

Data were analyzed at the cluster and individual participant levels. For continuous data, we reported numbers and means with standard deviations (SDs) or medians with interquartile ranges (IQRs) as appropriate. Categorical data were presented as frequencies with percentages.

As a feasibility trial, the study was not adequately powered to detect treatment group differences. However, data analyses were conducted using the intention‐to‐treat (ITT) principle for key effectiveness outcomes to provide initial estimates of treatment effects. Missing data were handled using multiple imputation methods. Imputation was based on participants′ age, sex, education, employment status, treatment group (intervention/control), and baseline measurement of the relevant variable. The resulting estimates were combined using Rubin′s method. Additionally, complete case analyses were conducted for both key and secondary effectiveness outcomes.

For continuous outcomes, results were presented in terms of cluster means. We used a mixed‐effects linear regression model that incorporated a random effect for centers to account for the cluster design and adjusted for both the individual baseline measurement of the relevant variable and the baseline mean of the relevant variable at the cluster level [41].

Binary outcomes were presented at the individual participant level, as the cluster sizes were small and calculating cluster proportions was not appropriate; the study was not designed to analyze binary outcomes at the cluster level. We compared treatment groups using a generalized linear mixed‐effects regression model with a logit link, including a random effect for centers to account for the clustered design, and adjusting for the individual baseline measurement of the relevant variable.

Effect estimates were presented as mean differences (MDs) with 95% confidence intervals (CIs) for continuous outcomes and odds ratios (ORs) with 95% CIs for binary outcomes. Additionally, we computed intraclass correlation coefficients (ICCs) with 95% CIs for key effectiveness outcomes at 6 months, both unadjusted and adjusted for individual baseline measurements, using random effects models. Data analyses were performed using Stata V.15.

### 2.9. Sample Size

In a feasibility trial, a formal sample size calculation is typically not required; however, it is recommended that each group have at least four clusters and a minimum of 50 participants per group for a valid analysis [42, 43]. In line with these recommendations and accounting for potential loss to follow‐up, the plan for this feasibility cluster RCT was to recruit a minimum of 14 clusters (seven per group) and 120 participants (60 per group).

### 2.10. Ethics and Other Related Issues

Ethics approval for the study was obtained from the Research Ethics Committee, Faculty of Medicine and Health Sciences, University of Nottingham, UK (511‐2003) and Ethical Review Board, NHRC, Nepal (66/2022). This feasibility trial was registered with a clinical trial registry. The study also received a clinical trial license from the Department of Drug Administration in Nepal. An independent Trial Steering Committee (TSC) comprising relevant experts monitored and provided overall supervision for the study.

## 3. Results

### 3.1. Trial Recruitment and Follow‐Up

**Figure 1:**
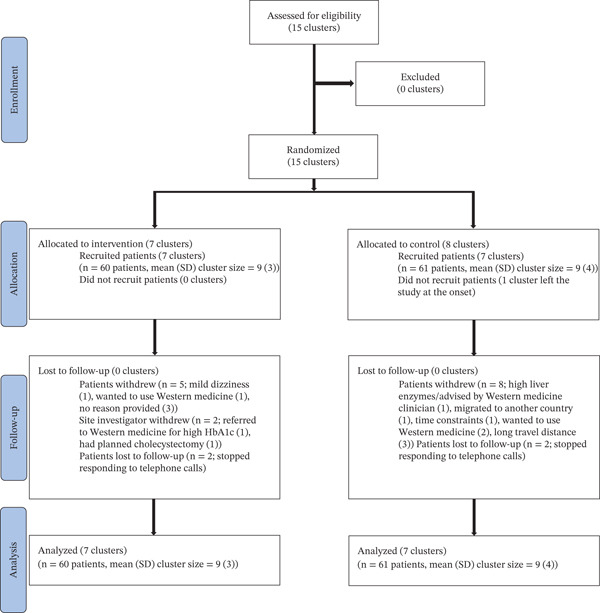
CONSORT flowchart.

Fourteen Ayurveda centers were approached, all of which were recruited into the study and completed it, with seven centers assigned to each group. Additionally, one center that was recruited did not enroll any participants and withdrew from the study at the outset (see Figure 1). The centers that completed the study were a mix of public and private (nine government and five private).

Out of 151 potential participants approached, 121 (80%) were recruited, with 60 in the intervention group and 61 in the control group. Of those recruited, 84% were followed up to 6 months (51 per group). It should be noted that one participant in the intervention group was withdrawn from the study by Ayurvedic practitioners, in accordance with the EB‐CPG recommendation for referrals (i.e., being referred to Western medicine for high HbA1c).

### 3.2. EB‐CPG Adherence in Intervention Group

The median adherence score to the EB‐CPG recommendations among the intervention group Ayurvedic practitioners ranged from 1 to 2 (indicating partial to adequate adherence) (see Table 1). All practitioners in the intervention group provided the EB‐CPG–recommended Ayurvedic antidiabetic medicine to participants; however, this was not always adequately documented in the case notes, resulting in a median score of 1.

**Table 1:** EB‐CPG adherence among intervention group Ayurvedic practitioners (*N* = 7).

	Median (interquartile range)
Type 2 diabetes mellitus diagnosis	2 (1–2)
Treatment targets	1 (1–1)
Lifestyle advice	
Diet	1 (1–2)
Exercise and physical activity	1 (1–1)
Alcohol intake	2 (1–2)
Smoking and drug misuse	2 (1–2)
Ayurvedic antidiabetic medicine	1 (0–2)
Complications screening and management	
Retinopathy	2 (1–2)
Foot problems	1 (1–2)
Diabetic kidney disease	1 (1–2)
Cardiovascular risk factors	1 (1–2)
Peripheral and autonomic neuropathy	2 (2–2)
Hyperglycemia management	^∗^
Intercurrent illness management	^∗^
Hypoglycemia management	^∗^
Advice on driving, insurance, fasting (including religious/sociocultural festivals), work, and holidays and travel	1 (1–2)

*Note:* 0 = not adhered to EB − CPG; 1 = partially adhered to EB − CPG; 2 = adequately adhered to EB − CPG.

^∗^Not applicable, as there were no cases.

### 3.3. EB‐CPG–Recommended Ayurvedic Antidiabetic Medicine Compliance in Intervention Group

The median (IQR) number of EB‐CPG–recommended Ayurvedic antidiabetic medicine capsules that participants in the intervention group did not consume over a 6‐month period was 0 (0–10). The median (IQR) number of days within those 6 months that they did not consume the medicine was 0 (0–2.5).

### 3.4. Ayurvedic Medicines Prescribed in Control Group

In the usual clinical practice group, a range of oral Ayurvedic medicines was prescribed by Ayurvedic practitioners (see Table S1), including both classical and proprietary formulations containing ingredients of plant, animal, or mineral origin—either individually or in combination, and in various forms and dosages.

### 3.5. Participants′ Characteristics

At baseline, the mean (SD) age of participants was 50.1 (10.4) years, and 48 (40%) were female. The mean (SD) HbA1c was 7.5% (0.8), fasting plasma glucose was 133.1 mg/dL (36.2), and HRQoL‐EQ‐5D index score was 0.9 (0.1). The two treatment groups were generally similar, with differences observed in a few variables, including household income, obesity, total cholesterol, low‐density lipoprotein, alcohol intake, anxiety, and illness perception (see Table 2). Participants lost to follow‐up were more likely to be male, less educated, employed, and have a mother tongue other than Nepali (see Table S2).

**Table 2:** Baseline characteristics in the study (at the individual participant and cluster levels).

	**Intervention**	**Control**
**Individual participant level**		
**Number of participants**	**60**	**61**
	**Mean (standard deviation) or median (interquartile range) or n/N (%)**	**Mean (standard deviation) or median (interquartile range) or n/N (%)**

Age (years)	50.5 (10.6)	49.8 (10.4)
Female sex	23/60 (38)	25/61 (41)
≥ Secondary school education	35/58 (60)	35/61 (57)
Employed	27/59 (46)	27/61 (44)
Gross monthly household income (Nepali rupee) ^∗^	40,000.0 (30,000.0–50,000.0)	30,000.0 (20,000.0–40,000.0)
Married	54/57 (95)	57/60 (95)
Nepali mother tongue	49/59 (83)	49/61 (80)
Hindu religion	54/58 (93)	61/61 (100)
Family history of diabetes (i.e., in any parent or sibling)	30/59 (51)	30/61 (49)
Existing self‐reported health conditions		
Obesity	6/59 (10)	12/61 (20)
Dyslipidemia	8/59 (14)	9/61 (15)
Hypertension	18/59 (31)	19/61 (31)
Coronary heart disease	1/59 (2)	0/61 (0)
Stroke	1/59 (2)	0/61 (0)
Peripheral arterial disease	0/59 (0)	1/61 (2)
Glycated hemoglobin (%)	7.2 (6.7–8.1)	7.4 (6.9–8.1)
Fasting plasma glucose (mg/dL)	136.2 (39.8)	130.2 (32.5)
Total cholesterol (mg/dL)	216.5 (50.9)	197.7 (46.2)
Low‐density lipoprotein (mg/dL)	121.8 (42.9)	95.3 (41.2)
High‐density lipoprotein (mg/dL)	46.5 (11.2)	49.0 (16.1)
Very low–density lipoprotein (mg/dL) ^∗^	38.0 (32.3–53.5)	35.0 (25.2–46.3)
Triglyceride (mg/dL)	187.5 (157.4–264.5)	182.0 (135.0–250.0)
Systolic blood pressure (mmHg)	128.5 (14.9)	132.4 (19.3)
Diastolic blood pressure (mmHg)	85.0 (10.3)	85.8 (10.6)
Heart rate (beats/min)	82.3 (11.0)	80.9 (12.2)
Weight (kg)	68.1 (10.0)	68.4 (9.9)
Body mass index (kg/m^2^)	27.2 (3.5)	27.6 (5.0)
Waist circumference (cm)	93.5 (89.0–99.0)	94.0 (89.0–100.0)
Regular high‐fat/deep‐fried food intake (i.e., ≥ 3 times/week)	22/58 (38)	28/60 (47)
≥ 5 portions of fruit and vegetables intake/day	13/58 (22)	10/60 (17)
Physical activity [44]		
Moderate to high	45/58 (78)	47/60 (78)
Total Metabolic Equivalent of Task (MET)‐min/week	1526.0 (693.0–4158.0)	1579.5 (742.5–4281.0)
Current tobacco usage	13/59 (22)	16/61 (26)
Current alcohol consumption	12/59 (20)	21/61 (34)
Health‐related quality‐of‐life [45, 46]		
EQ‐5D index score (< 0–1)	1.0 (0.9–1.0)	0.9 (0.8–1.0)
EQ‐5D visual analog scale score (0–100)	75.0 (65.0–90.0)	80.0 (60.0–85.0)
Mild to extremely severe depression, anxiety, and stress [47]		
Depression	1/59 (2)	3/61 (5)
Anxiety	1/59 (2)	12/61 (20)
Stress	0/59 (0)	1/61 (2)
Moderate to high threatening perception of illness [48]	12/59 (20)	19/59 (32)

**Cluster level (key effectiveness outcomes)**		
**Number of clusters**	**7**	**7**
**Mean (standard deviation) cluster size**	**9 (3)**	**9 (4)**
	**Mean (standard deviation) of cluster means**	**Mean (standard deviation) of cluster means**

Glycated hemoglobin (%)	7.4 (0.4)	7.7 (0.6)
Fasting plasma glucose (mg/dL)	136.1 (21.4)	126.1 (13.8)
Health‐related quality‐of‐life [45, 46]–EQ‐5D index score	1.0 (0.0)	0.9 (0.1)

*Note:* There were negligible missing data (≤ 4), except where indicated by an asterisk ( ^∗^). The numbers missing for these were as follows: gross monthly household income (*n* = 10) and very low–density lipoprotein (*n* = 18).

### 3.6. Participants′ Effectiveness Outcomes

In terms of key effectiveness outcomes, at the cluster level, HbA1c and fasting plasma glucose at 6 months were lower in the intervention group compared with the control group in both ITT and complete case analyses. However, these differences were not statistically significant (see Table 3). Similarly, the HRQoL‐EQ‐5D index score was higher in the intervention group but was also not statistically significant. ICCs for key effectiveness outcomes are reported in Table S3.

**Table 3:** Key effectiveness outcomes at 6 months (ITT and complete case analyses; at the cluster level).

	Intervention		Control		
	Number of individuals	Mean (standard deviation) of cluster means	Number of individuals	Mean (standard deviation) of cluster means	Mean difference (95% confidence interval) ^∗^
Glycated hemoglobin (%)					
Intention‐to‐treat analysis	60	6.8 (1.3)	61	7.0 (1.2)	−0.09 (−0.55, 0.38)
Complete case analysis	50	7.1 (1.1)	51	7.2 (0.8)	−0.17 (−0.68, 0.34)
Fasting plasma glucose (mg/dL)					
Intention‐to‐treat analysis	60	122.2 (36.4)	61	120.2 (32.9)	−1.86 (−18.80, 15.09)
Complete case analysis	50	133.4 (34.6)	50	122.6 (13.2)	−3.85 (−26.93, 19.24)
Health‐related quality‐of‐life [45, 46]–EQ‐5D index score (< 0–1)					
Intention‐to‐treat analysis	60	1.0 (0.1)	61	1.0 (0.2)	0.02 (−0.04, 0.07)
Complete case analysis	50	1.0 (0.0)	51	1.0 (0.1)	0.01 (−0.04, 0.06)

^∗^Clustering is allowed, and adjustments are made for the individual baseline measurement of the relevant variable and the baseline mean of the relevant variable at the cluster level.

Secondary effectiveness outcomes at 6 months are reported in Table S4. Compared with the control group, improvements were observed in the intervention group in several lifestyle and cardiovascular risk factors, among others, although these changes were not statistically significant.

### 3.7. Participants′ Safety

To ensure participants′ safety, we conducted liver and kidney function tests at baseline and 6 months (see Table S5). Isolated cases of AEs were reported (see Table S6). There was no SAE.

## 4. Discussion

To our knowledge, this is the first study to explore the use of an innovative approach involving EB‐CPGs in Ayurveda in general, and for the management of T2DM in particular. We conducted a feasibility cluster RCT in Nepal, which demonstrated promising results in the recruitment of Ayurveda centers and participants and their follow‐ups. In the intervention group, adherence to our EB‐CPG by Ayurvedic practitioners and compliance with the EB‐CPG–recommended Ayurvedic antidiabetic medicine by participants were encouraging. These findings indicate that it is feasible to undertake a definitive trial to evaluate the intervention′s effectiveness in T2DM management. Parameters estimated in this feasibility trial will be used to inform the design of the definitive cluster RCT. We also conducted semistructured qualitative interviews with practitioners and participants to explore their experiences and perspectives, including on trial recruitment and follow‐up, intervention adherence and medicine compliance, and challenges in the usual clinical care, which will help inform modifications to the intervention and definitive trial. This qualitative study is published elsewhere [49].

Compared with trials on Western medicine CPGs for managing T2DM conducted elsewhere [30, 31], recruitment and follow‐up in our feasibility trial in Nepal were relatively better and encountered no major hurdles. Some baseline differences likely arose due to chance variation, given the relatively small number of clusters randomized per arm. Additionally, self‐reporting may have contributed to some differences; for example, obesity as a self‐reported condition was perceived and reported differently between groups, although objectively measured body mass index and waist circumference at the study centers were similar. Additionally, participants showed promising compliance with the EB‐CPG–recommended Ayurvedic antidiabetic medicine. However, medication compliance in the intervention group was assessed through participant self‐reported diaries and capsule counts conducted at the study centers, both of which have inherent limitations. For instance, capsule counts may not accurately reflect true adherence, as participants could discard unused capsules to appear compliant. Nevertheless, these positive outcomes could be attributed to the strong preference for Ayurveda in Nepal, an indigenous system of medicine deeply intertwined with the culture and health beliefs of its people. The motivations for choosing Ayurveda over Western medicine and the diverse populations served by Ayurvedic practitioners in South Asia are well‐documented [8–13].

In this feasibility trial, one of the initial eligibility criteria was an HbA1c range of 6.5%–< 7.5%, which resulted in somewhat slower recruitment. Within the first 1.5 months of the trial, 89% (39 out of 44) of adults with newly diagnosed, treatment‐naïve T2DM were excluded due to an HbA1c of ≥ 7.5%. Following the initial months of good safety records, the upper limit was raised to < 9% after discussions with the TSC and approval from research ethics committees, which improved recruitment. As a feasibility trial, we were extra cautious, setting the lower cutoff arbitrarily for participant safety, without specific scientific evidence to support it. It is important to note that the definitive trial will not test a new drug but will be a pragmatic study addressing a real‐world issue, where individuals with high blood glucose levels continue to prefer Ayurveda, as observed in this feasibility study. Consequently, in the definitive trial, control group participants will continue to receive the usual clinical care, including commonly prescribed Ayurvedic formulations, whereas intervention group participants will receive the EB‐CPG–based care, including the EB‐CPG–recommended Ayurvedic antidiabetic medicine. Importantly, we will have a robust trial conduct and governance structure.

Systematic reviews and trials in Western medicine have demonstrated that CPGs can improve the quality of care, including the management of T2DM [30, 31, 50 **–** 52]. When evaluating improvements in care, it is essential to assess both process measures (e.g., adherence to guidelines) and outcome measures (e.g., health improvements), alongside structural elements, as emphasized in the Donabedian model [53]. In this feasibility trial, adherence to the EB‐CPG among Ayurvedic practitioners in the intervention group was encouraging. In line with findings from an umbrella review on strategies for implementing EB‐CPGs [54], we employed a variety of strategies to promote the uptake and adherence to our EB‐CPG. Although this feasibility trial was not powered to assess the intervention′s effectiveness, preliminary estimates suggest potential benefits across several outcomes, including key effectiveness measures such as HbA1c, fasting plasma glucose, and HRQoL, though none of which reached statistical significance. Importantly, no SAEs were reported. In this feasibility trial, the structural aspect was largely addressed by the resources provided. Looking ahead, our adequately powered definitive cluster RCT will determine whether practitioners′ use of our EB‐CPG effectively improves T2DM management in Nepal, particularly with respect to glycemic control. The definitive trial will also assess the proportion of participants achieving treatment targets [2]. Additionally, in this feasibility trial, participants were followed up for 6 months, which was a limitation due to the relatively short study duration. In the definitive trial, we plan to follow participants for at least 1 year, as T2DM is a long‐term condition [2].

Compared with the intervention group, where an evidence‐based, plant‐based Ayurvedic medicine was prescribed by Ayurvedic practitioners, practitioners in the control group routinely prescribed various nonevidence‐based Ayurvedic formulations for glycemic control in T2DM. These formulations commonly comprised ingredients of plant, animal, or mineral origin. This prescribing pattern aligns with findings from the existing literature [11, 13, 16, 17, 19–23, 25]. Many such formulations are also included in nonevidence‐based Ayurvedic CPGs for T2DM in India [7]. Our comprehensive systematic review and meta‐analysis included several of these formulations—such as *Berberis aristata*, *Emblica officinalis*, Hyponidd, shilajit, Swarnamakshika Bhasma, *Syzygium cumini*, and *Withania somnifera* [15, 35]. However, some were found to be ineffective for glycemic control in T2DM, whereas others require additional RCTs to establish their effectiveness and safety before they can be recommended. Furthermore, many of the Ayurvedic formulations routinely prescribed in clinical practice have not been evaluated in any RCT for glycemic control or safety in T2DM. The use of such nonevidence‐based formulations—particularly those containing mineral ingredients—may lead to serious adverse effects, including heavy metal toxicity [17, 21–25]. This underscores a significant gap between routine Ayurvedic clinical practice and the scientific evidence base. Addressing this gap presents opportunities for disinvestment, particularly for health policymakers and managers, by identifying and phasing out Ayurvedic formulations with no, minimal, or questionable value in T2DM. Resources could then be redirected from ineffective or unsafe options to those that are proven to be both effective and safe for glycemic control in T2DM. As we are planning the definitive trial and wish to minimize the risk of potential contamination in the future control group, we are unable to disclose the name of the Ayurvedic medicine prescribed to the intervention group at this early stage.

Ayurveda, now officially recognized or regulated in at least 17 countries, is gaining increasing global popularity [55]. Given that T2DM is a major global health concern, the EB‐CPG developed in our study is likely to generate interest not only in neighboring South Asian countries such as India, Sri Lanka, and Bangladesh—where Ayurveda is widely practiced—but also in countries with significant South Asian diaspora populations, particularly those in the Organisation for Economic Co‐operation and Development (OECD) [56]. As such, our study holds considerable potential for both regional and global impact. Furthermore, it plays a pivotal role in enhancing Ayurveda′s acceptance within scientific and broader communities, and in fostering its integration with Western medicine. Such an innovative approach involving EB‐CPGs could also be expanded to the Ayurvedic management of other chronic conditions, including additional cardiometabolic disorders.

In conclusion, this feasibility study demonstrated promising recruitment of Ayurveda centers and participants and their follow‐ups. In the intervention group, adherence to the EB‐CPG by Ayurvedic practitioners and participants′ compliance with the EB‐CPG–recommended Ayurvedic antidiabetic medicine were encouraging. Therefore, it is feasible to undertake a definitive cluster RCT that will evaluate the intervention′s effectiveness in T2DM management.

## Author Contributions

K.C. conceptualized and designed the study with input from P.G., V.U., M.H., B.B., N.T., S.K., S.M.G., T.K.B., P. J., J.L‐B., S.A.L., and M.D. K.C., S.K., P.R., P.G., V.U., B.B., and M.D. conducted the study. H.W. analyzed the data with support from S.A.L. and K.C. K.C. wrote the first draft of the manuscript, and all other authors contributed substantially to its revision.

## Funding

This study was funded by the UK′s Department of Health and Social Care, Foreign, Commonwealth and Development Office, Medical Research Council, and Wellcome Trust Joint Global Health Trials (MR/T003537/1). SABINSA provided the EB‐CPG–recommended Ayurvedic antidiabetic medicine for free. The authors express their sincere gratitude to them and the Department of Ayurveda and Alternative Medicine of the Government of Nepal. Special thanks go to the study participants, participating Ayurveda centers and Ayurvedic practitioners, data collectors, and members of the TSC.

## Disclosure

All authors read and approved the final manuscript. None of the funders were involved in the study design, data collection, management, analysis, interpretation, report writing, editing, or the decision to submit for publication.

## Conflicts of Interest

The authors declare no conflicts of interest.

## Supporting information


**Supporting Information** Additional supporting information can be found online in the Supporting Information section. Table S1: Ayurvedic medicines prescribed by control group Ayurvedic practitioners (*N* = 7). Table S2: Comparison of selected baseline characteristics between participants who were followed up at 6 months and those who were not followed up at 6 months (at the individual participant level). Table S3: ICC for key effectiveness outcomes at 6 months. Table S4: Secondary effectiveness outcomes at 6 months (complete case analyses; at the cluster and individual participant levels). Table S5: Liver and kidney function tests at baseline and 6 months (at the individual participant level). Table S6: AEs reported in the study.

## Data Availability

The dataset generated during this study is available from the corresponding author upon reasonable request.
