# Supplementary material for: Diabetes Management by Ayurvedic Practitioners Using a Clinical Guideline Versus Usual Practice: A Feasibility Cluster Randomized Trial in Nepal
Source: J Diabetes Res. 2026 Apr 29;2026:2602864. doi: 10.1155/jdr/2602864 (PMC13128970; doi:10.1155/jdr/2602864)
Supplement: Supplementary file 1 — Supporting Information Additional supporting information can be found online in the Supporting Information section. Table S1: Ayurvedic medicines prescribed by control group Ayurvedic practitioners (N = 7). Table S2: Comparison of selected baseline characteristics between participants who were followed up at 6 months and those who were not followed up at 6 months (at the individual participant level). Table S3: ICC for key effectiveness outcomes at 6 months. Table S4: Secondary effectiveness outcomes at 6 months (complete case analyses; at the cluster and individual participant levels). Table S5: Liver and kidney function tests at baseline and 6 months (at the individual participant level). Table S6: AEs reported in the study. [file JDR-2026-2602864-s001.docx]

Supplementary Table 1: Ayurvedic medicines prescribed by control group Ayurvedic practitioners (N=7).

| **Tablet** | **Capsule** | **Granule/**  **Powder** | **Syrup/**  **Decoction** | **Bhasma (Calx)** |
| --- | --- | --- | --- | --- |
| 1. Arogyavardhini Vati* 2. Chandraprabha Vati* 3. Chitrakadi Vati* 4. Debix 5. Diabecon 6. Diabecon DS 7. Glymin 8. Hyponidd 9. Madhu Kalp Vati 10. Madhumehari Yog (with gold) 11. Madhunashini Vati 12. Sarpgandhaghan Vati* 13. Vasant Kusumakar Ras* (with gold) 14. Zandiabts | 1. Amree Plus 2. Disoma 3. Glycoban 4. Shilajit* | 1. Diab Care 2. Gandhak* (mixed with other herbs) 3. Guduchi Sattva* 4. Jameda Churna 5. Madhumehari Granules 6. Nimbadi Churna* 7. Rohitakyadi Churna 8. Trikatu Churna* 9. Triphala Churna* 10. Single herbs: 11. *Azadirachta indica* 12. *Berberis aristata* 13. *Boerhavia diffusa* 14. *Curcuma longa* 15. *Emblica officinalis* 16. *Gymnema sylvestre* 17. *Momordica charantia* 18. *Swertia chirata* 19. *Syzygium cumini* 20. *Tinospora cordifolia* 21. *Trigonella foenum-graecum* 22. *Withania somnifera* | 1. Amrit Kadha 2. Amyron 3. Ojus | 1. Abhrak Bhasma* 2. Mukta Pishti* 3. Praval Panchamrit* 4. Swarnamakshik Bhasma* |

*Classical.

Supplementary Table 2: Comparison of selected baseline characteristics between participants who were followed up at 6 months and those who were not followed up at 6 months (at the individual participant level).

|  | **Followed up at 6 months** | **Not followed up at 6 months** |
| --- | --- | --- |
|  | **n/N (%) or mean (standard deviation)**  **or median (interquartile range)** | **n/N (%) or mean (standard deviation)**  **or median (interquartile range)** |
| **Number of participants** | **102/121 (84)** | **19/121 (16)** |
| **Number of participants in the intervention group** | **51/60 (85)** | **9/60 (15)** |
| **Number of participants in the control group** | **51/61 (84)** | **10/61 (16)** |
| Age (years) | 49.9 (10.5) | 51.5 (10.3) |
| Sex |  |  |
| *Male* | 59/102 (58) | 14/19 (74) |
| *Female* | 43/102 (42) | 5/19 (26) |
| Education |  |  |
| *≤Primary school* | 40/101 (40) | 9/18 (50) |
| *≥Secondary school* | 61/101 (60) | 9/18 (50) |
| Employment |  |  |
| *Employed* | 43/101 (43) | 11/19 (58) |
| *Others* | 58/101 (57) | 8/19 (42) |
| Gross monthly household income (Nepali rupee) | 32,000.0 (25,000.0-50,000.0) | 28,000.0 (20,000.0-40,000.0) |
| Marital status |  |  |
| *Married* | 92/98 (94) | 19/19 (100) |
| *Others* | 6/98 (6) | 0/19 (0) |
| Mother tongue |  |  |
| *Nepali* | 85/101 (84) | 13/19 (68) |
| *Others* | 16/101 (16) | 6/19 (32) |
| Religion |  |  |
| *Hindu* | 98/101 (97) | 17/18 (94) |
| *Others* | 3/101 (3) | 1/18 (6) |
| Glycated hemoglobin (%) | 7.5 (0.8) | 7.5 (0.8) |
| Fasting plasma glucose (mg/dL) | 132.2 (36.6) | 138.0 (34.7) |
| Health-related quality-of-life^45,46^ – EQ-5D index score (<0 to 1) | 0.9 (0.3-1.0) | 0.9 (0.6-1.0) |

Supplementary Table 3: ICC for key effectiveness outcomes at 6 months.

|  | **Intra-class correlation coefficient (95% confidence interval)** | |
| --- | --- | --- |
|  | **Unadjusted** | **Adjusted*** |
| Glycated hemoglobin | 0.22 (0.06, 0.55) | 0.12 (0.01, 0.58) |
| Fasting plasma glucose | 0.18 (0.03, 0.61) | 0.18 (0.03, 0.61) |
| Health-related quality-of-life^45,46^ – EQ-5D index score | 0.00 (0.00, 1.00) | 0.04 (0.00, 0.42) |

*Adjusted for the individual baseline measurement of the relevant variable.

Supplementary Table 4: Secondary effectiveness outcomes at 6 months (complete case analyses; at the cluster and individual participant levels).

|  | **Intervention** | | **Control** | |  |
| --- | --- | --- | --- | --- | --- |
|  | **Number of individuals** | **Mean (standard deviation) of cluster means or n/N (%)** | **Number of individuals** | **Mean (standard deviation) of cluster means or**  **n/N (%)** | **Mean difference or odds ratio (95% confidence interval)*** |
| **Continuous outcomes – cluster level** | | | | | |
| Total cholesterol (mg/dL) | 50 | 189.7 (36.0) | 50 | 179.8 (27.2) | 1.82 (-22.30, 25.94) |
| Low-density lipoprotein (mg/dL) | 50 | 103.0 (33.0) | 49 | 94.8 (18.5) | 0.23 (-18.49, 18.95) |
| High-density lipoprotein (mg/dL) | 50 | 43.2 (6.7) | 50 | 45.7 (10.0) | 4.54 (-0.60, 9.68) |
| Very low-density lipoprotein (mg/dL) | 49 | 41.1 (14.8) | 40 | 36.4 (7.1) | -4.90 (-16.29, 6.50) |
| Triglyceride (mg/dL) | 50 | 210.0 (64.6) | 49 | 191.0 (38.9) | -0.26 (-46.57, 46.05) |
| Systolic blood pressure (mmHg) | 50 | 125.5 (8.6) | 51 | 133.2 (12.3) | 2.69 (-4.14, 9.52) |
| Diastolic blood pressure (mmHg) | 50 | 83.1 (4.5) | 51 | 82.4 (6.3) | 0.17 (-4.07, 4.40) |
| Heart rate (beats/min) | 50 | 80.3 (3.7) | 49 | 80.1 (5.0) | 1.56 (-2.33, 5.45) |
| Weight (kg) | 50 | 68.8 (5.1) | 51 | 67.9 (8.9) | -0.19 (-1.97, 1.59) |
| Body mass index (kg/m^2^) | 50 | 27.1 (1.7) | 51 | 27.5 (2.9) | 0.10 (-0.73, 0.94) |
| Waist circumference (cm) | 50 | 92.3 (4.8) | 50 | 92.3 (12.7) | -0.30 (-2.63, 2.03) |
| Physical activity^44^ - Total MET-min/week | 50 | 1980.9 (1496.4) | 51 | 2662.0 (1341.3) | 278.36 (-1093.60, 1650.32) |
| Health-related quality-of-life^45,46^ - EQ-5D visual analog scale score (0 to 100) | 50 | 79.4 (7.7) | 51 | 80.8 (8.7) | -0.32 (-7.90, 7.26) |
| **Binary outcomes – individual participant level** | | | | | |
| High-fat/deep-fried food intake | 50 |  | 51 |  |  |
| *Never/occasionally* |  | 36/50 (72) |  | 35/51 (69) | Ref |
| *Regularly (i.e., ≥3 times/week)* |  | 14/50 (28) |  | 16/51 (31) | 1.16 (0.40, 3.31) |
| Fruit and vegetables intake | 50 |  | 51 |  |  |
| *<5 portions/day* |  | 47/50 (94) |  | 38/51 (75) | Ref |
| *≥5 portions/day* |  | 3/50 (6) |  | 13/51 (25) | 0.19 (0.05, 0.72) |
| Physical activity^44^ | 50 |  | 51 |  |  |
| *Low* |  | 12/50 (24) |  | 6/51 (12) | Ref |
| *Moderate/High* |  | 38/50 (76) |  | 45/51 (88) | 2.33 (0.64, 8.50) |
| Tobacco usage | 50 |  | 51 |  |  |
| *Never/past* |  | 38/50 (76) |  | 36/51 (71) | Ref |
| *Current* |  | 12/50 (24) |  | 15/51 (29) | 0.59 (0.10, 3.41) |
| Alcohol consumption | 50 |  | 51 |  |  |
| *Never/past* |  | 40/50 (80) |  | 43/51 (84) | Ref |
| *Current* |  | 10/50 (20) |  | 8/51 (16) | 0.20 (0.02, 2.47) |
| Depression^47^ | 50 |  | 51 |  |  |
| *Normal* |  | 47/50 (94) |  | 46/51(90) | Ref |
| *Mild/moderate/severe/extremely severe* |  | 3/50 (6) |  | 5/51 (10) | 1.46 (0.17, 12.39) |
| Anxiety^47^ | 50 |  | 51 |  |  |
| *Normal* |  | 48/50 (96) |  | 46/51(90) | Ref |
| *Mild/moderate/severe/extremely severe* |  | 2/50 (4) |  | 5/51 (10) | 0.67 (0.10, 4.31) |
| Stress^47^ | 50 |  | 51 |  |  |
| *Normal* |  | 50/50 (100) |  | 50/51 (98) |  |
| *Mild/moderate/severe/extremely severe* |  | 0/50 (0) |  | 1/51 (2) | Not calculable |
| Perception of illness^48^ | 50 |  | 51 |  |  |
| *Low threat* |  | 43/50 (86) |  | 40/51 (78) | Ref |
| *Moderate/high threat* |  | 7/50 (14) |  | 11/51 (22) | 0.63 (0.21, 1.84) |
| Satisfaction with treatment | 50 |  | 51 |  |  |
| *Very satisfied/satisfied* |  | 14/50 (28) |  | 11/51 (22) | Ref |
| *Very dissatisfied/dissatisfied* |  | 36/50 (72) |  | 40/51 (78) | 1.08 (0.21, 5.65)^ |

*Continuous outcomes: Clustering is allowed, and adjustments are made for the individual baseline measurement of the relevant variable and the baseline mean of the relevant variable at the cluster level. Binary outcomes: Clustering is allowed, and adjustment is made for the individual baseline measurement of the relevant variable.

^Only clustering is allowed, as it is impossible to collect treatment satisfaction data prior to the start of treatment. Reference: Hawthorne G, Sansoni J, Hayes L, et al. Measuring patient satisfaction with health care treatment using the Short Assessment of Patient Satisfaction measure delivered superior and robust satisfaction estimates. J Clin Epidemiol. 2014;67(5):527-37.

Supplementary Table 5: Liver and kidney function tests at baseline and 6 months (at the individual participant level).

|  | **Intervention** | | **Control** | |
| --- | --- | --- | --- | --- |
|  | **Baseline mean (standard deviation) or median (interquartile range)** | **6 months mean (standard deviation) or median (interquartile range)** | **Baseline mean (standard deviation) or median (interquartile range)** | **6 months mean (standard deviation) or median (interquartile range)** |
| **Liver function tests** | | | | |
| Albumin (g/dL) | 4.4 (0.3) | 4.2 (0.3) | 4.1 (0.3) | 4.2 (0.4) |
| Total bilirubin (mg/dL) | 0.8 (0.3) | 0.8 (0.2) | 0.8 (0.2) | 0.8 (0.2) |
| Direct bilirubin (mg/dL) | 0.2 (0.2-0.3) | 0.2 (0.1) | 0.2 (0.2-0.2) | 0.2 (0.1) |
| Indirect bilirubin (mg/dL) | 0.5 (0.2) | 0.5 (0.2) | 0.6 (0.2) | 0.6 (0.2) |
| Alkaline phosphatase (ALP) (U/L) | 150.9 (65.1) | 143.7 (66.9) | 142.2 (77.0) | 131.5 (74.0) |
| Alanine transaminase (ALT) (U/L) | 29.0 (21.8-48.5) | 26.8 (14.0-53.0) | 35.0 (25.0-42.9) | 33.0 (18.0-63.0) |
| Aspartate transaminase (AST) (U/L) | 26.0 (21.0-38.0) | 26.5 (18.0-40.0) | 32.5 (24.7-39.6) | 31.0 (16.0-59.2) |
| AST-ALT ratio | 1.0 (0.3) | 1.0 (0.3) | 1.0 (0.3) | 1.0 (0.2) |
| **Kidney function tests** | | | | |
| Sodium (mEq/L) | 139.5 (3.6) | 139.0 (3.3) | 140.6 (2.7) | 140.0 (2.5) |
| Potassium (mEq/L) | 4.0 (0.4) | 4.0 (0.3) | 4.2 (0.3) | 4.2 (0.3) |
| Urea (mg/dL) | 29.0 (7.7) | 28.2 (7.0) | 24.9 (5.2) | 25.6 (4.8) |
| Creatinine (mg/dL) | 0.9 (0.2) | 0.9 (0.2) | 0.8 (0.1) | 0.8 (0.2) |
| Urine albumin-to-creatinine ratio | 27.9 (31.2) | 20.8 (15.9) | 17.6 (10.8) | 16.3 (12.0) |

Supplementary Table 6: AEs reported in the study.

| **Intervention** | **Control** |
| --- | --- |
| - Belching and acid reflux - Burning sensation in soles - Early menstruation - Episodic weakness - High glycated hemoglobin - High lipids - High uric acid and bilateral knee pain - High urine albumin-to-creatinine ratio - Jaundice - Mild dizziness - Shortness of breath | - Abdomen pain - Burning sensation in the chest - Cold - Cough - Dengue - Dryness - Excessive micturition - Fever - Gastritis - Headache - High glycated hemoglobin - High lipids - High liver enzymes - Pain at the lipoma site - Tingling sensation (e.g., in lower limbs) |
